# Early life exposure to oestrogen and testicular cancer risk: evidence against an aetiological hypothesis

**DOI:** 10.1038/sj.bjc.6600246

**Published:** 2002-04-22

**Authors:** C C Hsieh, M Lambe, D Trichopoulos, A Ekbom, O Akre, H-O Adami

**Affiliations:** University of Massachusetts Cancer Center, 373 Plantation Street, Suite 202, Worcester, Massachusetts, MA 01605, USA; Department of Medical Epidemiology, Karolinska Institute, Stockholm, Sweden; Department of Epidemiology and Center for Cancer Prevention, Harvard School of Public Health, Boston, Massachusetts, MA 02115, USA

## Abstract

*British Journal of Cancer* (2002) **86**, 1363–1364. DOI: 10.1038/sj/bjc/6600246 www.bjcancer.com

© 2002 Cancer Research UK

## Sir

Testicular cancer has a peak incidence among men aged 25 to 34 years, suggesting early life influences (Akre *et al*, 1996). The hypothesis that exposure to endogenous or environmental oestrogenic compounds affects embryonic testis and increases the risk of testicular cancer has been put forth (Sharpe and Skakkeback, 1993). This intriguing hypothesis has acquired momentum and, in fact, was invoked as the main aetiologic mechanism in a recent major review (Dearnaley *et al*, 2001). If prenatal exposure to oestrogens were indeed important, one would expect that populations with higher levels of pregnancy oestrogens would experience a higher rate of testicular cancer. We have studied pregnancy hormone levels, including oestrogen levels, in two populations with very different rates of testicular cancer (Lipworth *et al*, 1999).

Adult pregnant women were recruited from maternity clinics of Beth Israel Hospital in Boston, MA, USA, and from hospitals affiliated with the Shanghai Medical University in Shanghai, China. Pregnant women were enrolled during their first prenatal visit to the collaborating maternity clinic. Eligibility requirements included that the pregnant women had to be less than 40 years old, had no more than one previous stillborn or liveborn child, took no hormonal medication during the index pregnancy and had no prior diagnosis of diabetes mellitus or thyroid disease. Three hundred and four Caucasian women in Boston and 335 Chinese women in Shanghai were studied. Pregnancy serum concentrations of oestradiol-17β (E2) and unconjugated oestriol (E3) were measured in maternal blood at weeks 16 and 27 of gestation. Details of the study have been published in this journal (Lipworth *et al*, 1999). Levels of E2 and E3 were consistently and significantly higher among Chinese women at both sample 1 and sample 2 (Table 1

**Table 1:**
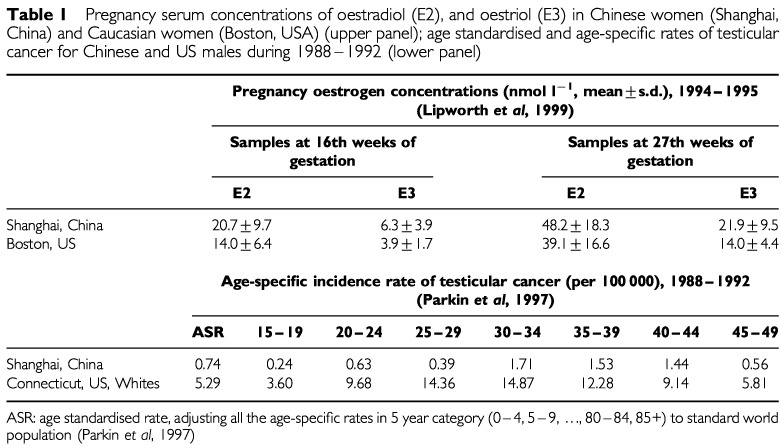
Pregnancy serum concentrations of oestradiol (E2), and oestriol (E3) in Chinese women (Shanghai, China) and Caucasian women (Boston, USA) (upper panel); age standardised and age-specific rates of testicular cancer for Chinese and US males during 1988–1992 (lower panel)

). The age-standardised rate as well as age-specific incidence rates of testicular cancer during the period of 1988–1992 for white and Chinese males (aged 15–49) were obtained from the Connecticut and the Shanghai Cancer Registries, respectively (Parkin *et al*, 1997). The rates for Chinese males in Shanghai at all ages were much lower than those for white males in Connecticut, despite evidence of exposure to higher levels of oestrogens *in utero*.

While the hypothesis linking high oestrogen exposure with testicular cancer and other disorders of the male reproductive system is ingenious, empirical support so far has been limited. Our data are incompatible with this hypothesis and, although they do not conclusively refute it, tend to reduce its plausibility.
